# A Review of Fixed Drug Eruption with a Special Focus on Generalized Bullous Fixed Drug Eruption

**DOI:** 10.3390/medicina57090925

**Published:** 2021-09-01

**Authors:** Hannah J. Anderson, Jason B. Lee

**Affiliations:** Department of Dermatology and Cutaneous Biology, Thomas Jefferson University, Philadelphia, PA 19107, USA; hannah.anderson@jefferson.edu

**Keywords:** fixed drug eruption, generalized bullous fixed drug eruption, Stevens-Johnson syndrome, toxic epidermal necrolysis, drug rash, FDE, GBFDE, SJS/TEN

## Abstract

Fixed drug eruption (FDE) is a cutaneous adverse drug reaction characterized by the onset of rash at a fixed location on the body each time a specific medication is ingested. With each recurrence, the eruption can involve additional sites. Lesions can have overlying vesicles and/or bullae, and when they cover a significant percentage of body surface area, the eruption is referred to as generalized bullous fixed drug eruption (GBFDE). Due to the widespread skin denudation that can be seen in this condition, GBFDE may be confused clinically with Stevens-Johnson syndrome/toxic epidermal necrolysis (SJS/TEN). While treatments described for GBFDE include supportive care, topical and/or systemic steroids, and, recently, cyclosporine, the mainstay of management involves identifying and discontinuing the causative drug. This review article will provide an overview of FDE with an emphasis on its generalized bullous variant.

## 1. Introduction

Fixed drug eruption (FDE) was first described in 1889 by Bourns, and the term fixed drug eruption, or “éruption érythémato-pigmentée fixe” was coined by Brocq in 1894 [1,2]. Brocq described “round or oval apparently edematous plaques, which varied in size from that of a coin to that of a palm; and which recurred on various parts of the body. As the eruption faded, there remained in the affected areas, a pigmentation of variable shades and duration” [1]. While other variants have since been identified, this depiction of FDE as a localized, pigmented eruption that is subject to recurrence remains true today.

## 2. Presentation

**Figure 1:**
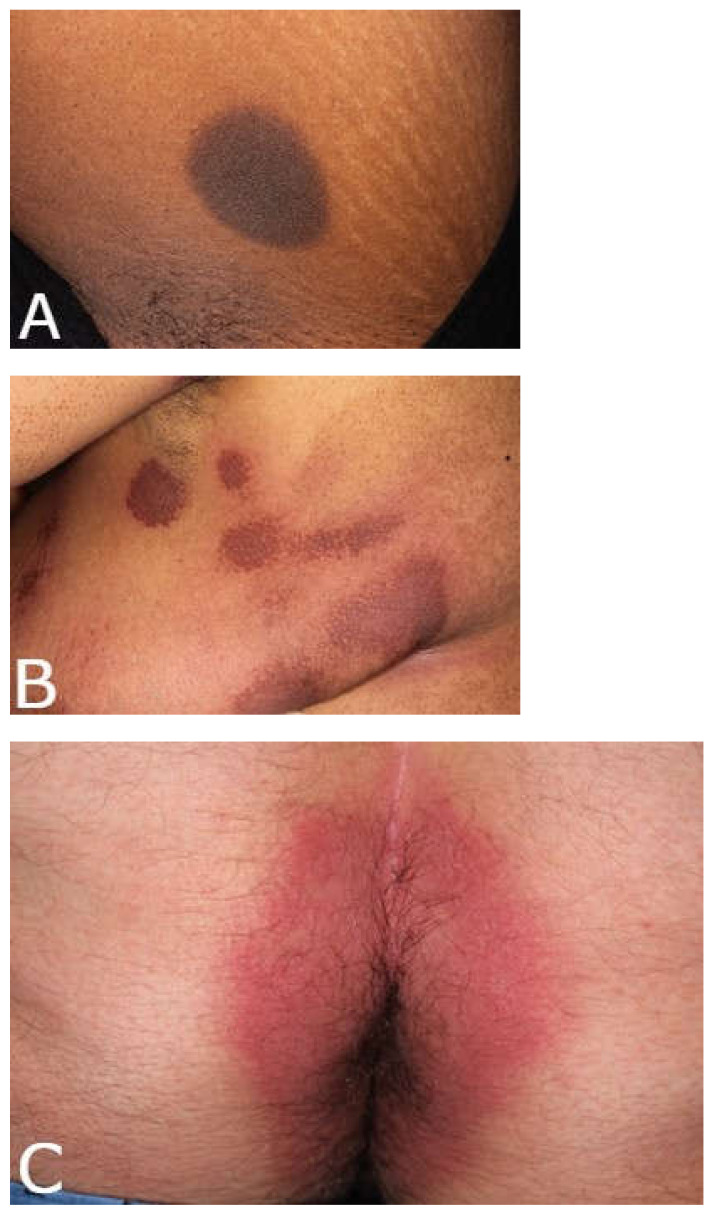

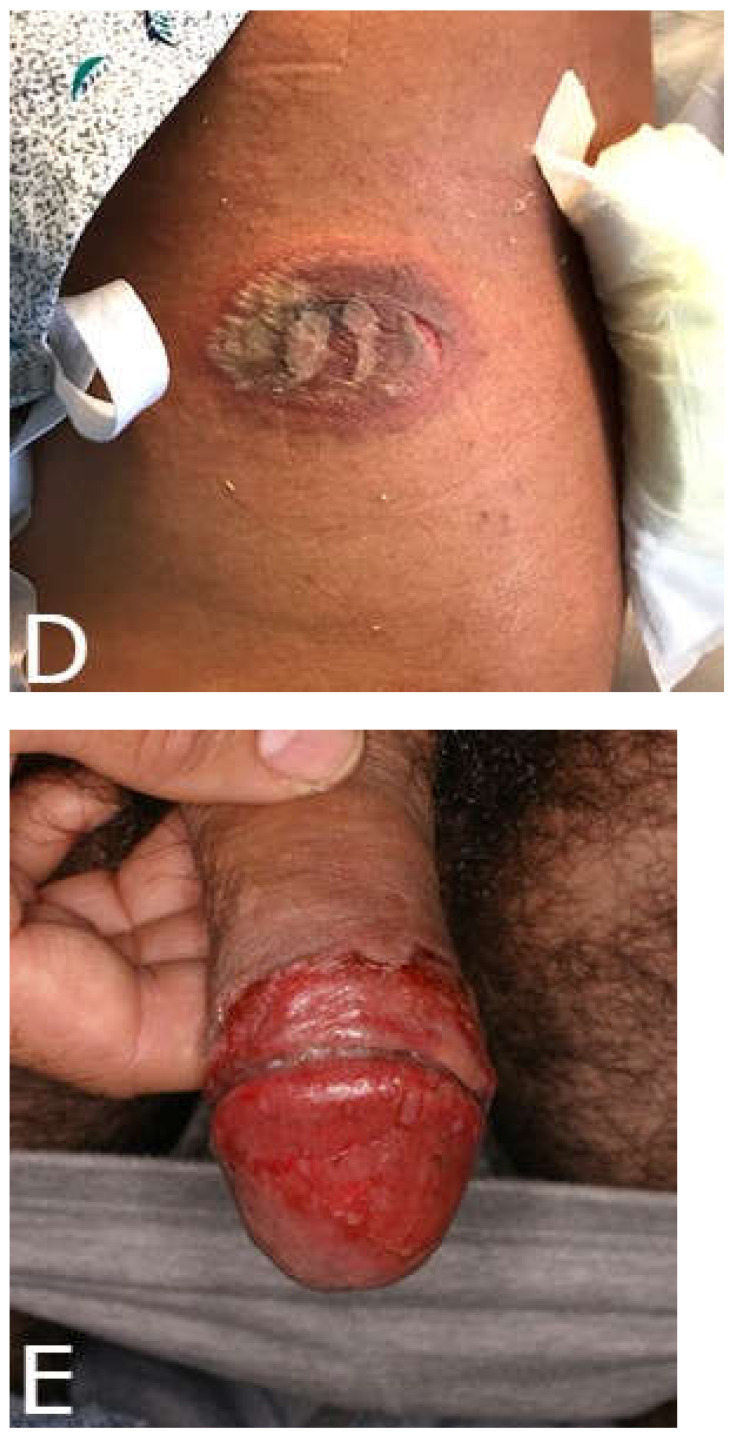
Examples of fixed drug eruptions (FDE). (**A**–**C**) Non-bullous FDE with the classic morphology of erythematous to violaceous, round to oval patches that may have a dusky center. (**D**,**E**) Examples of bullous/erosive FDE.

FDE is defined by the same-site recurrence of a rash each time a medication is ingested. With each additional exposure to the offending medication, the lesions can increase in size and number of sites involved [3]. The typical morphology of FDE is a solitary, well-demarcated erythematous to violaceous, round to oval patch with a dusky center (Figure 1a–c) [4]. After the acute inflammation has resolved, post-inflammatory hyperpigmentation lasting weeks to months typically remains. The lesions may present as blisters, vesicles, and/or bullae, that rupture easily, leaving erosions or shallow ulcers (Figure 1d,e) [2]. In one retrospective study of 57 patients with FDE in Southern India, one-third were found to have bullous and erosive lesions [5]. In some cases, there is an extensive eruption of bullae in addition to the characteristic patches of FDE, a condition referred to as generalized bullous fixed drug eruption (GBFDE) [2]. One study of bullous FDEs presenting to a dermatology department in Tunisia over an 18-year period found that 44.4% of cases were localized and 55.6% of cases were generalized [6].

Another variant of FDE is the nonpigmenting subtype, which heals without residual pigmentation changes after two to three weeks [7]. This variant has historically been considered rare and is classically associated with pseudoephedrine [7]. However, a 2010 study of 59 cases of FDE found that 20% were of the nonpigmenting subtype, and the authors found no relationship between the clinical subtype and the implicated medication [8]. This suggests that the nonpigmenting variant is more common and associated with a greater number of drugs than previously thought.

The most commonly reported site of FDE also varies depending on the study. The upper extremities were reported as the most common site in one study [9], while others have reported the lips [3,5] as the most frequent site. One study reported a sex-dependent distribution of lesions, with 89% of women presenting with limb involvement (especially on the hands and feet), whereas 90% of men had lesions on the genitals [8]. Mucous membranes are frequently affected. One study found that 24.2% of FDE cases had genital involvement [10], while another study found that the oral mucosa was affected in 34.7% of established cases of FDE [11]. In one retrospective study, oral mucosal lesions were found to accompany genital lesions in 68.8% of cases of genital FDE [11]. Lesions of the oral mucosa were most commonly bullous and erosive; however, an aphthous or erythematous morphology was observed in a minority of patients, which may lead to a misdiagnosis of Behçet’s disease [11]. About five percent of patients with FDE have involvement of the mucous membranes alone without accompanying cutaneous lesions [8].

FDE can be solitary, scattered, or generalized; the majority of patients have five or fewer lesions [3,9]. The interval between drug exposure and the onset of FDE can be as long as two weeks, but most patients, especially those who have been exposed to the medication on prior occasions, develop the eruption within 48 h [5,8,9,10]. At the time of presentation, the majority of patients note a history of similar lesions in the past [9,10]. With each recurrence, lesions characteristically appear in the same site(s) as prior eruptions, and with repeated exposure to the causative medication, involvement can spread to additional sites that were not previously involved [10,11]. About one-quarter of patients experience local symptoms such as itching and/or burning in association with the eruption [3].

## 3. Epidemiology

FDE can occur in all ages, including children and the elderly, but it most commonly occurs in young- to middle-aged adults, with reported median ages ranging between 35 and 60 [5,6,8,9,10,12]. The average age of patients with non-generalized bullous FDE was found to be significantly younger than that of GBFDE patients (47.2 versus 69.1), and the median ages were similarly disparate (46 versus 74) [12]. FDE occurs essentially equally in men and women [6,10,12].

## 4. Pathogenesis

FDE is mediated by CD8+ memory T cells that reside in the basal layer of the epidermis of resting FDE lesions [13]. Within 24 h of ingestion of a culprit medication, these CD8+ T cells migrate upward in the epidermis [13], produce cytokines such as interferon-gamma and TNF-alpha [14,15], and take on the phenotype of a natural killer cell, expressing the cell surface molecule CD56 as well as the cytotoxic molecules granzyme B and perforin [13]. This activity leads to the epidermal necrosis that is observed in FDE [15]. At the same time, CD4+ Foxp3+ regulatory T cells migrate into the epidermis, curbing the damage inflicted by the CD8+ T cells [13]. The action of the CD4+ regulatory T cells, which includes the production of the anti-inflammatory cytokine IL-10, explains the self-limited nature of FDEs [15]. After the acute phase of FDE has resolved, the CD8+ cells lose the natural killer phenotype that they had gained during an acute flare of FDE, and they remain quiescent in the basal layer of the epidermis at the site of prior eruption for many years [13].

FDEs to the same drug have been reported in immediate family members, indicating that there may be a genetic component to the pathogenesis of FDE [16,17,18,19,20,21,22]. Medications implicated in familial cases of FDE include tetracycline/demeclocycline [16], feprazone [17,18], trimethoprim/sulfamethoxazole [19,20], diphenhydramine and aspirin [21], and ibuprofen [22]. Specific associations have been found between human leukocyte antigen (HLA) genes and FDE from certain drugs. For example, the HLA-A30 B13 Cw6 haplotype was found to be significantly more frequent in patients with FDE secondary to trimethoprim/sulfamethoxazole than healthy control patients [20], while the HLA-B22 allele is associated with feprazone-induced FDE [17,23].

## 5. Associated Agents

A multitude of substances has been implicated in FDE. The most common causative drugs differ depending on the geographic area. In one retrospective analysis of FDEs in a three-year period in France, the most common etiologic agent was acetaminophen, followed by NSAIDs like piroxicam, naproxen, and ibuprofen [8]. A study over a 14-year period in Tunisia demonstrated NSAIDS as the drug category most commonly associated with FDE, followed by antibiotics, especially amoxicillin, levofloxacin, and doxycycline [9]. An analysis of 450 cases in Pakistan revealed trimethoprim/sulfamethoxazole as the causative agent in 73% of cases [3]. Antiepileptics such as phenytoin, carbamazepine, and phenobarbital are frequently implicated [5,9]. Agents other than medications, such as intravenous contrast and the influenza vaccine, have also been reported to cause FDE [24,25].

Foods have also been implicated in fixed eruptions that present similarly to FDE, and this condition has been termed “fixed food eruption” (FFE). FFEs have been reported from a variety of foods, including tree nuts such as cashew nuts, almonds, and walnuts; seafood such as shell fish and crab; fruits including strawberries and kiwi; and lentils [26,27]. Quinine in tonic water has been associated with FFE [28]. Yellow food color additives such as tartrazine and Quinoline Yellow that are commonly found in foods and medications have been implicated in so-called “fixed food-and-drug eruption [26,29].” Another example of fixed food-and-drug eruption was reported with lactose as the causative agent [30]. The patient presented with recurrent lesions on the bilateral eyelids after exposure to dairy products and four unrelated drugs that each contained lactose as an inactive ingredient [30]. Lactose was confirmed as the implicated substance by oral challenge test [30]. Patients without a suggestive medication history who present with lesions resembling FDE should be asked about any association between the cutaneous eruption and types of foods consumed.

## 6. Diagnosis

**Figure 2:**
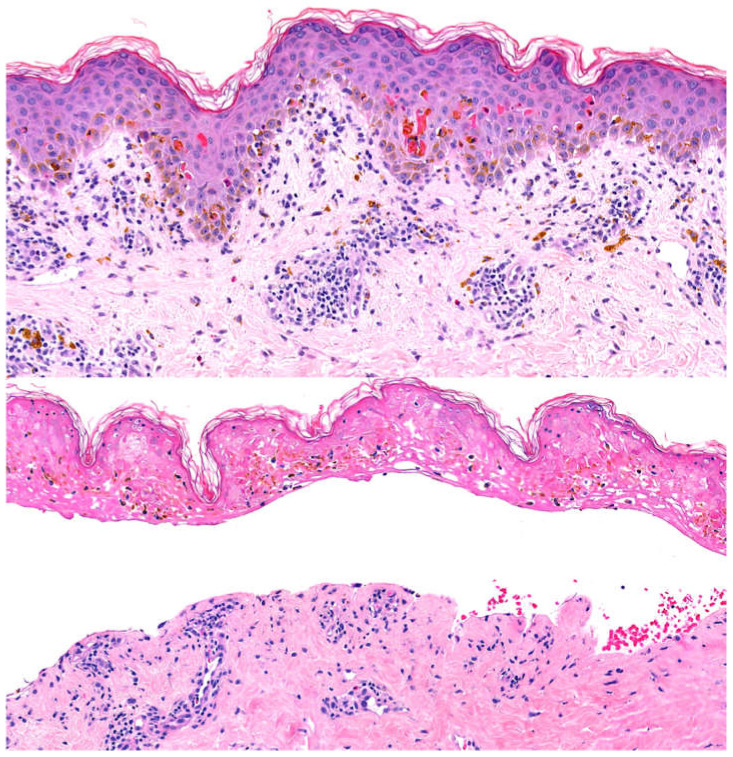
Characteristic histology of FDE shows vacuolar interface dermatitis, necrotic keratinocytes, and pigment incontinence (upper). Full thickness necrosis results in a subepidermal blister in acute rapidly evolving lesions (lower).

The diagnosis of FDE can often be made on clinical grounds based on distinctive appearance and history of a similar eruption with drug exposure. However, when the presentation is ambiguous, especially in variants of FDE such as GBFDE or the nonpigmenting subtype, a biopsy may be performed. Histopathologically, FDE is characterized by vacuolar interface dermatitis with both superficial and deep perivascular infiltration of eosinophils and lymphocytes [31,32]. Individual necrotic keratinocytes can be seen scattered throughout the epidermis, and pigment incontinence is typical [32]. In cases of FDE that have recurred in the same site, fibrosis of the papillary dermis is sometimes present in addition to many melanophages [31]. Clinically and histologically, the differential diagnosis for FDE may include other cutaneous eruptions characterized by vacuolar interface dermatitis such as erythema multiforme, bullous graft-versus-host disease, and Stevens-Johnson syndrome/toxic epidermal necrolysis (SJS/TEN), and histological findings must be correlated with the clinical picture [31]. Figure 2 demonstrates the characteristic histology of FDE.

Once the diagnosis of FDE is established, it is important to attempt to identify the causative drug. Because FDE can become increasingly severe with each recurrence, the patient should avoid the culprit medication, as well as cross-reacting substances, once it is recognized [4]. Sometimes, the patient is able to pinpoint a prescription or over-the-counter drug that was started shortly prior to the onset of the rash. However, in many cases, the causative medication is unclear. In this case, diagnostic testing to identify the etiologic agent can be performed.

The oral challenge test, also known as the oral provocation test, was traditionally the diagnostic gold standard [9]. This method has higher sensitivity than other diagnostic studies such as patch testing [9]. The oral challenge test is performed by administering a fraction, typically one-tenth, of a therapeutic dose of medication and assessing for recurrence of the rash [33]. The dose administered may be increased if the initial test dose does not produce a reaction, and the administration of a full therapeutic dose without a response constitutes a negative result [9]. This test is contraindicated in known cases of generalized FDE [4,6] and is rarely performed today, even when patients only have a history of localized reactions, due to the risk of instigating GBFDE [34]. When an oral challenge test is performed, it is done so only under close physician surveillance [34].

A recent case series proposed new guidelines for safely performing oral challenge tests, even in patients with a history of generalized FDE [35]. For patients who presented with less than three lesions of FDE, the authors recommended starting with the standard average daily dose and, if no reaction is seen, increasing to twice the daily dose [35]. If twice the daily dose does not result in a reaction, the drug is excluded as the cause of FDE. For patients who presented with more than three FDE lesions or had oral involvement, the authors recommended a graded oral challenge [35]. The graded challenge starts with 20% of the daily dose and increases by ten percent every 30 min until the patient develops lesions of FDE or a cumulative dose of twice the average daily dose is reached [35]. This case series was limited to two patients, however, and one of them developed new FDE lesions with the graded oral challenge test in addition to recurrence of FDE at prior sites of involvement [35]. Further studies will need to be done to determine the safety of these proposed guidelines.

Patch testing is considered a safer, albeit less sensitive, method of elucidating the causative drug in FDE [34]. Patch tests are performed at the site of a previous lesion of FDE at least two weeks after the resolution of a prior eruption [33]. The medication is diluted in petrolatum or water at a concentration of 10 to 20 percent [33]. The patch is applied for 24 to 48 h, and infiltrated erythema or an intense local reaction constitutes a positive test result [34]. A retrospective review of 52 patients with a clinical diagnosis of FDE who underwent patch testing demonstrated a positive reaction in lesional skin in 40.4% of patients [34]. The positive reactivity in this study was almost exclusive to NSAIDs, whereas other drug classes, particularly antibiotics, consistently gave negative test results, even when clinical suspicion was high [34]. In the same study, patch testing in non-lesional skin was negative in all but one patient [34]. The fact that diagnostic utility depends on the implicated medication class is a limitation of patch testing.

The lymphocyte transformation test (LTT) is used rarely to confirm diagnosis of FDE. This assay involves incubating a patient’s peripheral blood mononuclear cells (PBMCs) with the suspected culprit drug and measuring their proliferation rate compared to the patient’s unexposed PBMCs [36,37]. A stimulation index of greater than 1.8 to 2.0 is considered positive [36,37]. While this test is generally unrevealing for the diagnosis of FDE, it has been shown to be confirmatory for cases of FDE caused by etoricoxib, allopurinol, fluconazole, and tranexamic acid [36,37,38,39]. The LTT may be most useful when clinical suspicion is high that a case of FDE is caused by a drug known to have a high false-negative rate in lesional patch tests, such as allopurinol [34].

## 7. Generalized Bullous Fixed Drug Eruption (GBFDE)

GBFDE has been defined as typical FDE lesions as well as blisters and erosions involving at least ten percent of the body surface area and at least three of six different anatomic sites (specifically, the head and neck, anterior trunk, back, upper extremities, lower extremities, and genitalia) [32]. Due to the widespread distribution, dusky coloration, and skin detachment seen in GBFDE, this condition is often confused clinically with SJS/TEN [40]. In fact, Alan Lyell, the first author to describe SJS/TEN in a case series of four patients, recanted his report 34 years later, stating that two of the four cases had actually been GBFDE [41,42].

### 7.1. Diagnosis and Distinction from SJS/TEN

Some distinctions between GBFDE and SJS/TEN can be made on clinical grounds, although features of these conditions can have significant overlap. Patients with GBFDE tend to be older and are less likely to have constitutional symptoms than patients with SJS/TEN [12]. While mucosal involvement was traditionally thought to be less frequent and less severe in GBFDE, bullous or erosive lesions of the mucosa in GBFDE are frequently observed [43]. One retrospective study of a single referral center in northern Taiwan found that 66.7% of patients with GBFDE had mucosal involvement, versus 30% of cases of non-generalized bullous FDE [12]. GBFDE always presents within one to two weeks (but most frequently within 48 h) of ingestion of the causative medication [5,8,9,10], while latency between drug exposure and clinical presentation of SJS/TEN is most commonly one to three weeks [44]. The skin lesions of SJS/TEN tend to coalesce and may have atypical targets, while the patches and bullae of GBFDE tend to be well-demarcated and have larger areas of normal skin in between lesions [43,45]. GBFDE heals with hyperpigmentation but no permanent scarring, whereas SJS/TEN is associated with significant scarring, especially on mucosal sites [45]. A history of a similar, albeit possibly less severe, skin eruption in response to the culprit drug can often be elicited in cases of GBFDE [5].

A skin biopsy may be performed to confirm the diagnosis of GBFDE when the clinical presentation is ambiguous. The biopsy specimen should include an area of lesional intact epidermis and a portion of the blister or denuded area; thus, a shave biopsy that includes a broader area of the lesion may be more optimal than a 4 to 6 mm punch biopsy. Characteristic histopathologic findings of GBFDE consist of a subepidermal blister or denuded epidermis and vacuolar alterations at the dermo-epidermal junction, with a variable number of necrotic keratinocytes within lesional intact epidermis. Though the infiltrate of inflammatory cells is variable, there is usually a brisk, moderately dense perivascular infiltrate of lymphocytes and interstitial eosinophils. In response to the necrosis of the epidermis, a variable number of neutrophils may also be present. Unfortunately, similar findings are observed in bullous erythema multiforme and in acute bullous graft-versus-host disease [46]. In contrast, SJS/TEN, particularly TEN, is more commonly characterized by a near absence of or sparse inflammatory infiltrate and broad epidermal necrosis [46].

A retrospective analysis found that SJS/TEN demonstrates clustering of apoptotic keratinocytes, especially at the edge of the blister in the plane between the epidermis and dermis [32]. In GBFDE, clustering was not typically seen, and the necrotic keratinocytes were instead scattered throughout the epidermis [32]. Another study comparing SJS/TEN and GBFDE replicated this finding, identifying the so-called “fire flag sign” (more than two aggregated dyskeratotic keratinocytes in the epidermis) in 100% of cases of SJS/TEN and 0% of cases of GBFDE [12]. Infiltration of eosinophils is more commonly found in cases of GBFDE, and when it is seen, the eosinophils tend to be more abundant in number [12,32]. In one study, pigment incontinence was seen in 100% of cases of GBFDE versus 33.3% of cases of SJS/TEN [12]. Unfortunately, GBFDE and SJS/TEN cannot always be distinguished on histopathologic basis alone as they share overlapping findings. Thus, clinical-pathologic correlation remains the gold standard in establishing the diagnosis of GBFDE [31].

Expression levels of various immunohistochemical markers differ between GBFDE and SJS/TEN. In one study, the number of dermal CD4+ T cells and dermal Foxp3+ regulatory T cells was significantly greater in GBFDE, whereas intraepidermal CD56+ cells were seen more frequently in SJS/TEN [32]. While the cytotoxic molecules Fas, FasL, perforin, and granzyme B did not differ between the two conditions, the number of intraepidermal cells expressing granulysin, which is known to be a major mediator of epidermal necrosis in SJS/TEN, was shown to be significantly greater in SJS/TEN than GBFDE [32]. On the other hand, a retrospective histopathological analysis of six types of adverse cutaneous drug reactions found a high rate of granulysin expression in SJS, TEN, and FDE, with positivity in 93%, 88%, and 100% of cases, respectively [47]. Mild-to-moderate epidermal granulysin expression was seen at a higher rate in SJS and TEN than in FDE (69% of cases of SJS, 85% of cases of TEN, and 45% of cases of FDE); however, intense expression was seen in 18% of FDE cases versus 0% of SJS and TEN cases [47]. The contradictory findings regarding epidermal granulysin expression indicate that histologic detection of this molecule may not be able to differentiate SJS/TEN and FDE.

Serum granulysin levels have been found to be significantly lower in GBFDE compared to SJS/TEN [32], leading some authors to advocate the use of a serum granulysin test as a method to rapidly diagnose SJS/TEN [44]. In fact, an immunochromatographic test to detect high levels of serum granulysin was developed that gave positive results in 80% of patients with SJS/TEN compared to only four percent of patients with “ordinary drug-induced skin reactions” [48]. However, the percentage of GBFDE patients who would test positive with this assay has not been studied. The level of granulysin expressed in blister fluid in cytotoxic T cell-mediated disorders, including SJS/TEN and both generalized and localized bullous FDE, has been found to be significantly higher than in non-cytotoxic T cell-mediated blistering disorders such as bullous lupus erythematosus, pemphigus vulgaris, and bullous pemphigoid [49]. Moreover, blister granulysin levels in SJS/TEN have recently been shown to be significantly higher than in bullous FDE, although the comparison did not differentiate between localized and generalized bullous FDE [49]. Further studies will be necessary to determine if lesional granulysin levels within blister fluid can help to differentiate SJS/TEN and GBFDE; however, these entities are ultimately clinical diagnoses based on consistent history and physical exam findings.

### 7.2. Prognosis

GBFDE is generally thought to be associated with a much better prognosis than SJS/TEN. A retrospective study published in 2012 called this belief into question when it found no significant difference in mortality rates for patients with GBFDE compared to patients with SJS/TEN when matched for age and extent of skin detachment [43]. The overall mortality rate for GBFDE was 22% [43]. However, the patients with GBFDE who were included in this study were all initially reported to a database as potential cases of SJS/TEN prior to this diagnosis being ruled out, and almost one-third of the GBFDE patients had mucous membrane involvement of at least two sites [43]. The patients in this study may have represented a more severe sample of GBFDE cases, and given the favorable outcome that is frequently reported in this entity, more studies will need to be conducted investigating the mortality rate of GBFDE. To date, there have been no other studies corroborating these results.

### 7.3. Treatment

In general, the treatment for FDE is the identification and discontinuation of the culprit medication [50]. This is also the mainstay of therapy for GBFDE [44]. There have been numerous reports of patients with GBFDE whose skin findings resolved with discontinuation of the causative drug and supportive care alone [1,40,44,51,52]. However, topical steroids [53,54] as well as short courses of systemic steroids [1,55,56,57,58,59,60,61,62], most commonly oral prednisone or prednisolone, are often used to treat GBFDE. Rarely, GBFDE requires transfer to a burn intensive care unit for aggressive wound care [24]. Due to increasing reports of severe cases of GBFDE, including the aforementioned study describing a high mortality rate in this condition [43], there has been recent interest in the use of cyclosporine for treatment. Thus far, there have been six cases of GBFDE treated with cyclosporine described in the literature [63,64,65,66,67]. Five of the cases were in adults, and these patients were treated with five to 14 days of cyclosporine, typically at doses of three or five mg/kg daily, with resolution of erythema and cessation of further blistering [63,64,65,66]. One pediatric case of GBFDE was treated with five mg/kg cyclosporine divided into two daily doses for one week followed by 2.5 mg/kg/day for another two weeks [67]. In this patient, improvement of erythema and cessation of further blistering were noted within 24 h of cyclosporine therapy [67]. There are no clinical trials comparing the efficacy of supportive care alone versus treatments such as topical steroids, systemic steroids, or cyclosporine for GBFDE. It is unclear if these interventions hasten the resolution of the eruption or decrease mortality compared to discontinuation of the etiologic drug alone.

## Data Availability

Not applicable.
